# Reduced Dopamine Transporter Availability and Neurocognitive Deficits in Male Patients with Alcohol Dependence

**DOI:** 10.1371/journal.pone.0131017

**Published:** 2015-06-29

**Authors:** Che-Hung Yen, Yi-Wei Yeh, Chih-Sung Liang, Pei-Shen Ho, Shin-Chang Kuo, Chang-Chih Huang, Chun-Yen Chen, Mei-Chen Shih, Kuo-Hsing Ma, Giia-Sheun Peng, Ru-Band Lu, San-Yuan Huang

**Affiliations:** 1 Graduate Institute of Medical Sciences, National Defense Medical Center, Taipei, Taiwan, ROC; 2 Department of Neurology, Tri-Service General Hospital, National Defense Medical Center, Taipei, Taiwan, ROC; 3 Department of Psychiatry, Tri-Service General Hospital, National Defense Medical Center, Taipei, Taiwan, ROC; 4 Department of Psychiatry, Beitou Branch, Tri-Service General Hospital, National Defense Medical Center, Taipei, Taiwan, ROC; 5 Department of Psychiatry, Taipei Branch, Buddhist Tzu Chi General Hospital, Taipei, Taiwan, ROC; 6 Department of anatomy and biology, National Defense Medical Center, Taipei, Taiwan, ROC; 7 Institute of Behavior Medicine, College of Medicine, National Cheng Kung University, Tainan, Taiwan, ROC; Chiba University Center for Forensic Mental Health, JAPAN

## Abstract

Dopamine plays an important role in the development of alcohol dependence, cognitive dysfunction, and is regulated via dopamine transporter activity. Although dopamine transporter activity is critically involved in alcohol dependence, studies observing this relationship are limited. Thus the current study examined whether dopamine transporter availability is associated with developing of alcohol dependence and cognitive dysfunction. Brain imaging with ^99m^Tc-TRODAT-1 as a ligand was used to measure dopamine transporter availability among 26 male patients with pure alcohol dependence and 22 age- and sex- matched healthy volunteers. The Wisconsin Card Sorting Test (WCST) and Tridimensional Personality Questionnaire (TPQ) were administered to assess neurocognitive functioning and personality traits, respectively. Compared to healthy controls, patients with alcohol dependence showed a significant reduction in dopamine transporter availability (p < 0.001), as well as diminished performance on the WCST (p < 0.001). Dopamine transporter availability was negatively correlated with both total and perseverative WCST errors among healthy controls, but only patients with alcohol dependence showed a positive correlation between dopamine transporter availability and a harm avoidance personality profile. Thus, reductions in dopamine transporter availability may play a pathophysiological role in the development of pure alcohol dependence, given its association with neurocognitive deficits. Moreover, personality may influence the development of pure alcohol dependence; however, additional clinical subgroups should be examined to confirm this possibility.

## Introduction

Alcohol dependence (AD) is a heterogeneous mental disorder that is confounded by several factors [1]. Studies have shown that changes in central dopaminergic neurotransmission can influence drinking behaviors in both animals and humans [2], and dysfunction of central dopaminergic system is an important factors in AD pathogenesis. The dopamine transporter (DAT) is a trans-membrane protein [3, 4] responsible for the reuptake of dopamine from the synaptic clefts into the presynaptic terminal. DAT knockout mice show a reduction in the intra-neuronal vesicular storage pool of dopamine and a profound elevation of extracellular dopamine levels [5]. Hence, evaluating central DAT availability may help better understand the state of dopaminergic neurons in the brain.

DAT has been suggested as the molecular site of action for reinforcing mechanisms related to drug addiction [6]. Alcohol is highly addictive, and long-term alcohol abuse may impair the brain’s dopaminergic system. However, research using live neuroimaging of DAT among patients with AD has been limited until now. One postmortem study observed reduced DAT availability in the nucleus accumbens related to late-onset alcoholism [7]. Additionally, previous brain imaging studies using single-photon emission tomography (SPECT) have shown a striatal DAT reduction among late-onset alcoholics [8, 9]. However, a relationship between striatal DAT reduction and alcohol dependence has not been seen in previous studies using positron emission tomography (PET) [10, 11]. These conflicting results could likely be attributed to the fact that AD is a complex disorder, and that the presence of different clinical subtypes may influence DAT availability. Thus, assessing a homogenous AD group may provide better insight into the association between striatal DAT availability and chronic alcoholism.

Previous research has shown reduced prefrontal cortical dopamine transmission among patients with AD [12]; these areas include association cortices implicated in attention and executive function. Neurocognitive deficits in memory, attention, and other domain have been associated with AD in previous studies [13]. The Wisconsin Card Sorting Test (WCST) is a well-known test for assessing working memory and cognitive flexibility [14]. Although healthy volunteers with higher striatal DAT density than patients tend to perform better on the WCST [15], using the WCST data to explore differences in striatal DAT availability among AD subjects has been limited. Therefore, it is important to determine whether the WCST can effectively detect any relationship between striatal DAT availability and neurocognitive deficits among alcohol abusers.

Dopamine is implicated in the regulation of specific personality traits [16, 17], and dopamine transporter/receptor availability may also be associated with certain traits [18, 19]. However, the relationship between DAT availability and personality has not been clearly established either among healthy subjects or patient populations [19, 20]. Thus, the current study investigated striatal DAT density and its association to both cognitive functioning and specific personality traits among patients with pure AD compared with healthy controls. We hypothesized that subjects with pure AD and healthy controls would show differences in striatal DAT availability and WCST performance and that striatal DAT availability would correlate with WCST scores. We further investigated a putative association between personality traits and dopaminergic neuronal activity in the human brain.

## Materials and Methods

### Sample preparation

This study enrolled male participants only because evidence suggests that gender is a significant confounding factor in research on striatal DAT availability [21]. The Institutional Review Board for the Protection of Human Subjects at the Tri-Service General Hospital (TSGH), a medical teaching hospital within the National Defense Medical center in Taiwan. The protocol was approved by the Ethics Committee of TSGH for the Protection of Human Subjects (TSGHIRB No. 099-05-017) and the methods were carried out in accordance with the approved guidelines. All participants were informed about the aims and other details of the study, and provided their written, informed consent. Subjects who voluntarily sought treatment for alcohol dependence at the TSGH between 2009 and 2013 and met inclusion/exclusion criteria were eligible to participate. All participants were older than 20 years of age. The patients had been diagnosed with alcohol dependence based on the Diagnostic and Statistical Manual of Mental Disorders, fourth edition (DSM-IV-TR), and were not dependent on other substances. Each patient and control were initially evaluated by an attending psychiatrist and then interviewed by a well-trained psychologist, using the Chinese version of the Modified Schedule of Affective Disorder and Schizophrenia-Lifetime (SADS-L) to screen out other psychiatric conditions. Subjects were excluded if they had a co-morbid axis I or II mental disorder (except AD), medical conditions that could alter cerebral functioning, head trauma involving loss of consciousness, or any neurological disease. The alcohol group consisted of 26 subjects with pure AD recruited during intoxication or withdrawal, but last drinking time less than 48 hours previously. Of the psychotropics, only lorazepam (2–8 mg/day) was permitted, to prevent alcohol withdrawal before the imaging study. SPECT studies were performed within the first week of recruitment. The control group consisted of 22 physically and psychiatrically healthy male volunteers. No participants were taking medications that could affect the central dopamine system during the period of the study.

### Imaging acquisition, processing, and data analysis

The procedure for preparing ^99m^Tc-TRODAT-1 for use in the imaging protocol has previously been described in detail [22, 23]. The brain SPECT scan with ^99m^Tc-TRODAT-1 was carried out within one week of the patient’s last alcoholic drink. TRODAT-1 kits were provided by the Institute of Nuclear Energy Research (Taiwan). Images were acquired 4 h after administration of a single bolus of 740 MBq (20 mCi) of ^99m^Tc-TRODAT-1 via antecubital vein. At the start of the scan, participants were placed in the camera in a supine position and the head fixed with a head holder. Fifteen dynamic images of the brain were acquired using a dual-headed gamma camera (Helix SPX; Elscint, Haifa, Israel) equipped with ultrahigh resolution fan-beam collimators (HUFB-75). Total acquisition time was 30 min. Data were acquired in a 128 × 128 matrix through 360° (180° for each sense of rotation) with rotations at 3° intervals at 30 s per angle step, with a pixel size of 3.17 × 3.17 mm (in a 1.4 zoom) and slice thickness of 3.4 mm. An external digital camera was used to monitor patients in order to prevent head movement during scanning, and sinograms and linograms were used for internal imaging quality checks.

Images were reconstructed using a back projection method with a Metz filter. Attenuation correction was performed using Chang’s first-order method. The SPECT images were analyzed along the level of the canthomeatal line. The composite image from the three highest-activity basal ganglia slices from a given participant was co-registered with the corresponding CT image to exclude possible brain lesions, and to delineate standardized regions of interest (ROIs) for the caudate, putamen, and striatum. MRI was used to recheck the brain lesions if the CT demonstrated inconclusive results. The defined ROIs were then manually applied to the other SPECT slices for that subject [21]. The occipital cortices (OC), which had low DAT concentrations, were also drawn in the same way and served as background areas. The uptakes of ^99m^Tc-TRODAT-1 in various brain regions was measured 4 h after injection, and the specific uptake ratio (SUR) of each region was calculated by the following equation: (*ROI*_*target*_—*ROI*_*reference*_ */ROI*_*reference*_). The researcher drawing ROIs on the images was blinded to subject group.

### Wisconsin Card Sorting Test

An experienced psychologist administered a computer-aided WCST on the same day as the SPECT scan. During the WCST, all participants were asked to match response cards with four stimulus cards along one of three perceptual dimensions (color, form, or number). Verbal feedback (right or wrong) was given without revealing information regarding the dimensions. After ten consecutive correct matches, the classification principle changed without warning, demanding flexibility in set shifting. There were 128 response cards during the test, and the test proceeded until six sorting categories had been acquired or until all the cards had been sorted. In accordance with the WCST manual [24], the following parameters were analyzed: (1) total corrects; (2) total errors; (3) perseverative errors; (4) non-perseverative errors; (5) categories completed; and (6) failure to maintain set. Evidence suggests that education and gender do not significantly influence performance on the WCST among Taiwanese subjects [25].

### Tridimensional Personality Questionnaire

We used the Chinese version of the Tridimensional Personality Questionnaire (TPQ). The Chinese version of TPQ is a self-administered, true-false instrument that excludes the reward-dependence dimension, which does not have adequate reliability among Han Chinese in Taiwan (Cronbach's α = 0.54) [26]. We measured two dimensions having acceptable internal consistency, namely novelty seeking (32 items, Cronbach's α = 0.72) and harm avoidance (34 items, Cronbach's α = 0.89).

### Statistical Analysis

Group differences on demographic characteristics with normally distributed continuous variables (age and education) were analyzed using Student’s t-test for independent samples, and non-normally distributed continuous variables (e.g. SUR for each brain region, WCST performance and TPQ performance) were analyzed using Mann Whitney U tests. As this study was based on several multiple comparisons, results could have arisen due to Type I errors. Therefore, Bonferroni corrections were applied to reduce issues related to family-wise error rates. Given that each participant had three correlated SURs (caudate, putamen, and striatum), a multiple linear regression using the generalized estimating equation (GEE) was implemented to adjust clustering within individuals. The effects of specific factors on the SURs were assessed with an exchangeable working correlation structure.

Spearman’s rank correlations were carried out to examine the association between striatal DAT availability and the WCST and TPQ parameters. Correlations between the SURs and other parameters (e.g. age, daily alcohol intake, severity, and year of AD) were assessed using Pearson correlations. All statistics were analyzed using SPSS software version 19.0 for Windows (SPSS Inc., Chicago, IL, USA). An analysis was considered statistically significant if its associated p-value was less than or equal to 0.05 (two-tailed).

## Results

A total of 48 male individuals participated, including 26 patients with AD (mean age 42.73 ± 10.43 years) and 22 healthy controls (mean age 39.64 ± 9.10 years). Each subject underwent SPECT scanning, and completed the WCST and TPQ. Subjects characteristics are presented in Table 1. There were no age differences between patients with AD and healthy controls (t = 1.435, p = 0.158). However, patients with AD had fewer years of education compared with healthy controls (t = -3.689, p = 0.001).

**Table 1:** Demographic Characteristics and Parameters of Wisconsin Card Sorting Test in People with Pure Alcohol Dependence and Healthy Controls.

	Pure Alcohol dependence (*n* = 26)	Healthy control (*n* = 22)		
	Mean ± SD	Mean ± SD	*t*	*p* ^a^
Age (y)^a^	42.73 ±10.43	39.64 ± 9.10	1.435	0.158
Education(y)^a^	13.15 ± 3.33	16.64 ± 3.17	-3.689	0.001
Duration of alcohol use (y)	12.15 ± 8.12	n/a		
Wisconsin Card Sorting Test			*z*	*p* ^b^
Total corrects ^b^	66.50 ± 17.49	70.68 ± 5.95	- 0.746	0.456
Total errors^b^	45.96 ± 26.86	19.86 ±15.23	-3.572	< 0.001
Perseverative errors ^b^	21.92 ± 15.79	9.73 ± 6.76	-3.367	0.001
Non-perseverative errors ^b^	27.92 ± 22.10	10.14 ± 9.24	-3.490	< 0.001
Categories complete ^b^	3.35 ± 2.37	5.59 ± 1.18	-3.623	< 0.001
Failure to maintain set ^b^	1.12 ± 1.34	0.73 ± 1.03	-0.837	0.402
Tridimensional Personality Questionnaire (TPQ)			*z*	*p* ^b^
Novelty seeking ^b^	15,08 ± 4.65	12.95 ± 3.63	-1.723	0.085
Harm avoidance ^b^	14.88 ± 5.55	11.82 ± 4.68	-1.973	0.049

^a^ Independent samples *t*-tests.

^b^ Mann-Whitney *U* test.

Patients with AD showed significant impairment on several WCST measures, including total errors (z = -3.572, p < 0.001), perseverative errors (z = -3.367, p = 0.001), non-perseverative errors (z = -3.490, p < 0.001), and categories completed (z = -3.623, p < 0.001). These results were significant after Bonferroni correction (Conservative p value would be 0.05/10 = 0.005). In contrast, we found no significant differences on the total number of correct trials, or a failure to maintain set (Table 1). In term of personality trait, results showed marginal differences between patients with AD and healthy controls on novelty seeking (NS) and harm avoidance (HA) (p = 0.085 for NS; p = 0.049 for HA).

**Fig 1:**
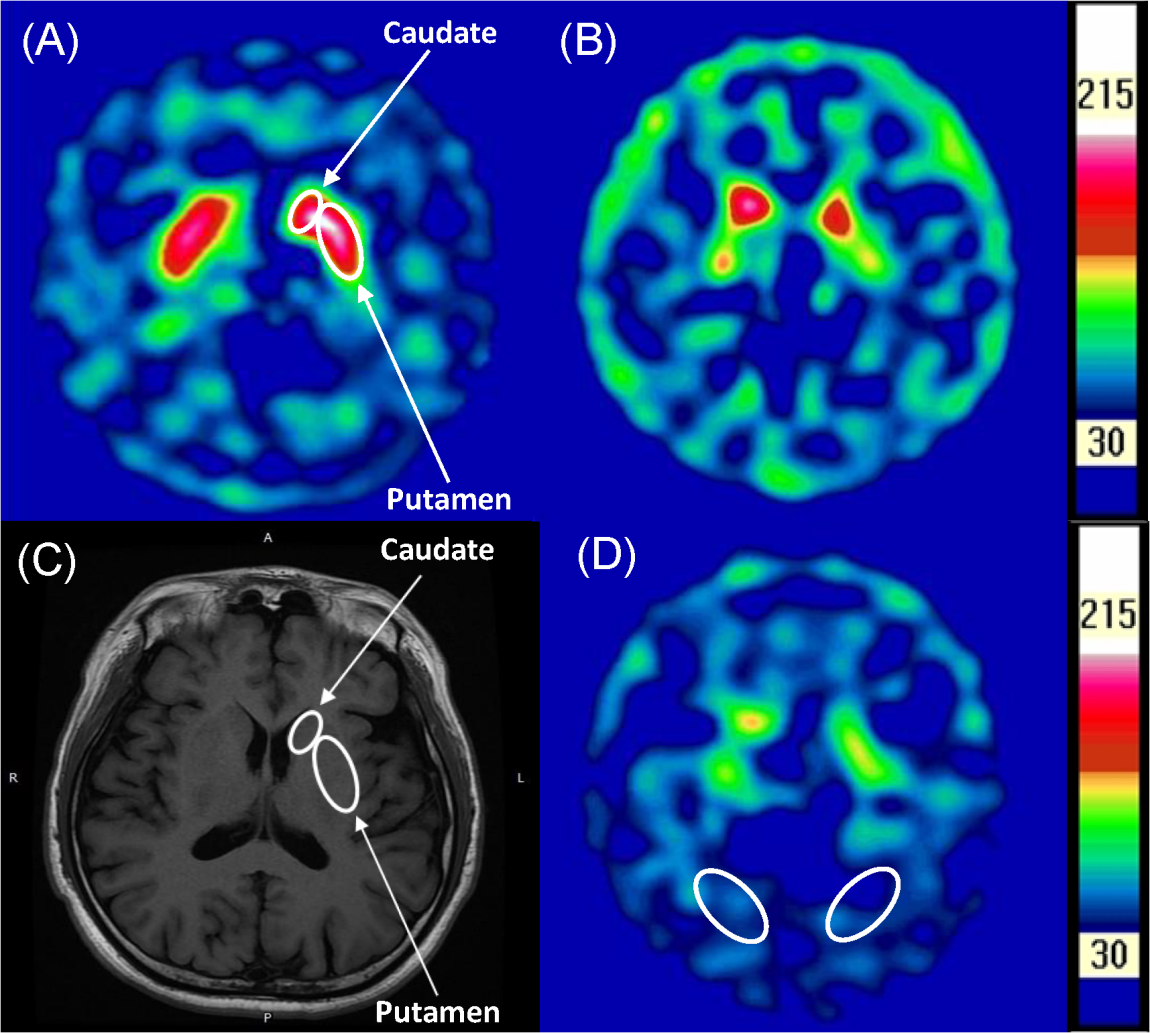
^99m^Tc-TRODAT-1 with single photon emission computed tomographic images of a control subject (A) in comparison with an age matched alcohol-dependent patient (B) in transverse slices at the level of striatum, and the corresponding MRI for the control subject (C). Regions of interest shows are for caudate and putamen (A and C), and for occipital lobe which is the reference region (D).

**Fig 2:**
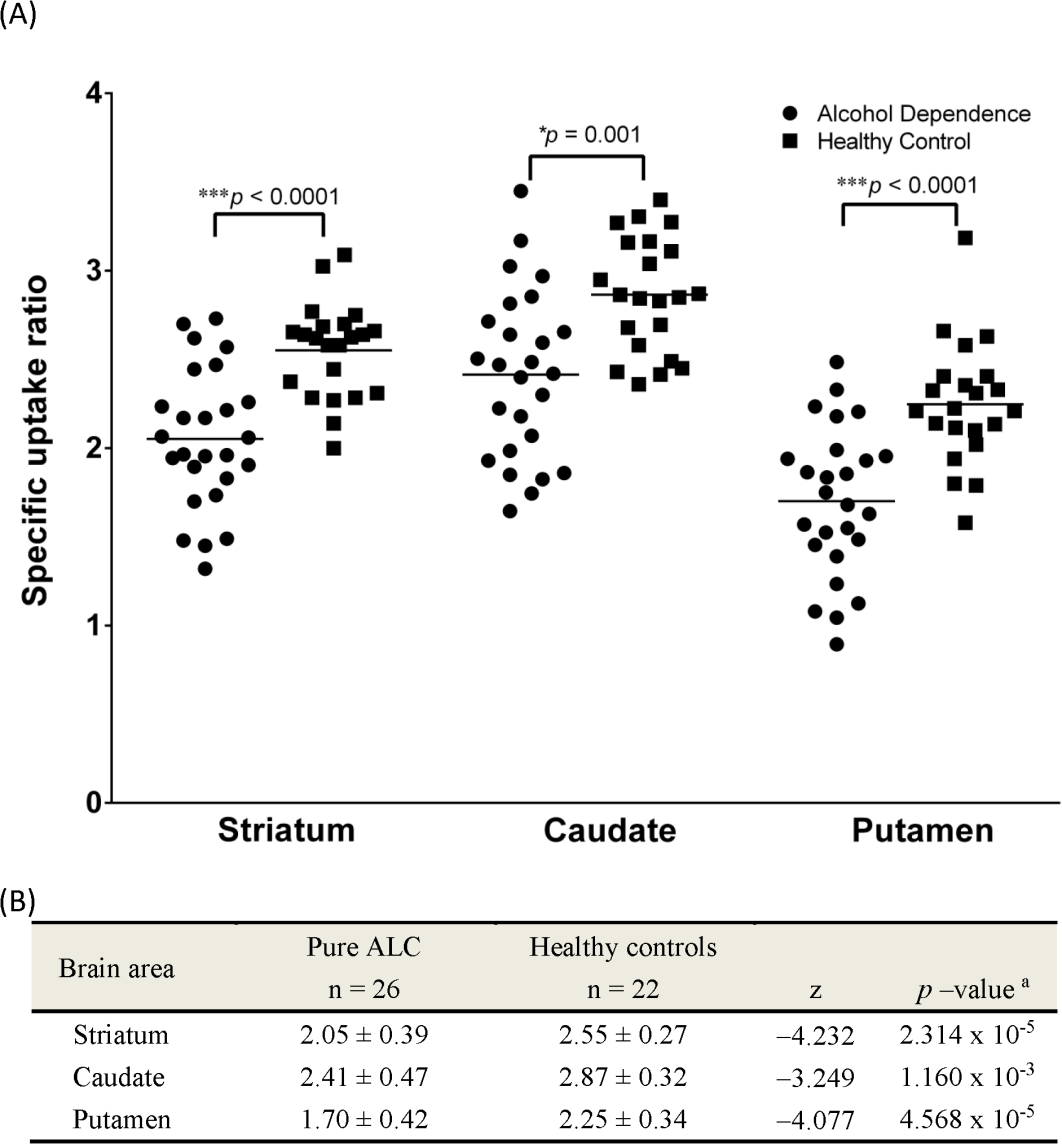
A scatter plot (A) and general data (B) of specific uptake ratio (SUR) of DAT in striatum, caudate and putamen calculated from ^99m^Tc-TRODAT-1 SPECT in alcohol dependent subjects and healthy controls. Horizontal bars indicates mean value of SUR. (mean ± SD). ^a^ *p* value of Mann-Whitney *U* test.

^99m^Tc-TRODAT-1 images of a patient with AD and a healthy control are shown in Fig 1. Individual with pure AD showed decreased radio signals with ill-defined margins in the striatum compared to healthy control. For the pure AD group, SUR values across the whole striatum, putamen, and caudate were 2.05 ± 0.39, 1.70 ± 0.42, and 2.41 ± 0.47, respectively. In the healthy control group, the corresponding values were 2.55 ± 0.27, 2.25 ± 0.34, and 2.87 ± 0.32, respectively. Patients with AD had significantly lower SURs in all three brain regions (striatum, putamen and caudate, p = 0.001 and ≤ 0.001, respectively; in Fig 2A and 2B). There were no significant difference in DAT availability between left and right brain regions (including the caudate, putamen and striatum, separately) in both subjects groups (S1 Table). The generalized estimating equation (GEE) analysis was performed to explore the effects of variables such as age, smoking status, education, and group on the SURs. The caudate and putamen SURs differed significantly from the striatum (p < 0.001). When analyzing the correlation between age and SUR, a significant effect was found (p < 0.001), but education years and smoking status did not have any significant association with SUR (Table 2). Since healthy controls did not have histories of alcohol abuse, only patients with AD were analyzed regarding correlations between SUR, daily alcohol intake, severity, and year of AD. A significant negative correlation between striatal SUR and year of AD (P< 0.001) was observed, but no significant associations emerged between SURs and daily alcohol intake and severity.

**Table 2:** The effects of age, education years, smoking status, brain regions, and groups on the SUR using the GEE method.

Variable	Estimate	SE	χ^2^	*df*	*p*
Age	0.029	0.004	46.581	1	<0.001
Education years	0.012	0.009	1.905	1	0.167
Total caudate vs. total striatum	0.321	0.025	168.934	1	<0.001
Total putamen vs. total striatum	0.320	0.025	163.083	1	<0.001
Nonsmoking vs. Smoking	0.165	0.113	2.142	1	0.143
Numbers of cigarettes smoked per day	0.003	0.002	1.203	1	0.273
Pure AD vs. controls	0.466	0.103	20.576	1	<0.001

AD: Alcohol dependence; SE = standard error, *df* = degree of freedom.

**Fig 3:**
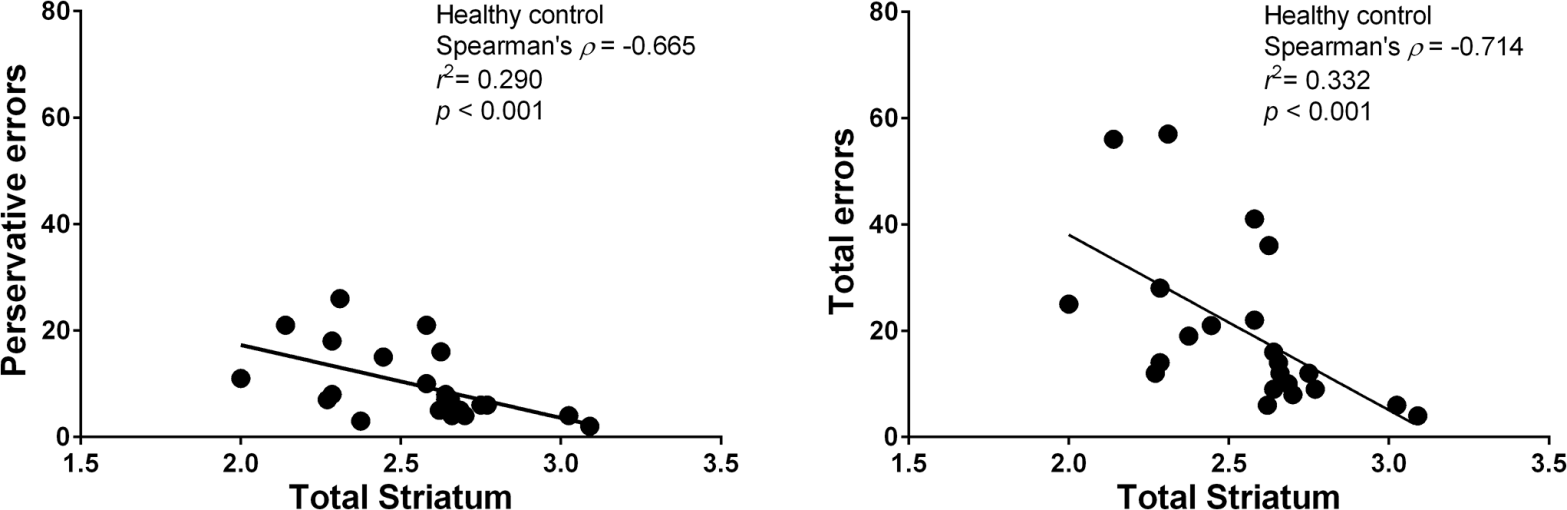
Graph showing correlation of striatal specific uptake ratio of [^99m^Tc] TRODAT-1 with perseverative errors and total errors. Significant association between these parameters existed healthy controls.

The association between striatal SUR and WCST parameters among healthy controls revealed that total correct (rho = -0.732, p < 0.001), total errors (rho = -0.714, p < 0.001), and perseverative errors (rho = -0.665, p < 0.001) reached statistical significance, and a marginal association was observed for non-perseverative errors (rho = -0.596, p = 0.003). Patients with AD had a marginally significant association between striatal SUR and total errors (rho = -0.445, p = 0.023), perseverative errors (rho = -0.416, p = 0.035), and categories completed (rho = -0.392, p = 0.048). However, these results did not meet the significance threshold after adjusting for multiple comparisons (Conservative P value would be 0.05/36 = 0.0014) (Table 3). Fig 3 presents the significant associations found between striatal SUR and total and perseverative errors among healthy controls.

**Table 3:** Association between WCST parameters and DAT availability in pure AD patients and healthy controls using Spearman’s rank correlation.

WCST Parameter	Pure AD (*n* = 26)			Healthy controls (*n* = 22)		
	*rho* coefficiency (*p* value)			*rho* coefficiency (*p* value)		
	striatum	caudate	putamen	striatum	caudate	putamen
TC	0.028(0.893)	0.016(0.937)	0.084(0.684)	0.732(<0.001)	0.579(0.005)	0.506(0.016)
TE	0.445(0.023)	0.280(0.166)	0.490(0.011)	0.714(<0.001)	0.548(0.008)	0.569(0.006)
PE	0.416(0.035)	0.285(0.158)	0.495(0.010)	0.665(<0.001)	0.670(0.001)	0.356(0.104)
NPE	0.275(0.174)	0.175(0.393)	0.277(0.171)	0.596(0.003)	0.375(0.086)	0.570(0.006)
CC	0.392(0.048)	0.233(0.252)	0.461(0.018)	0.365(0.095)	0.268(0.228)	0.342(0.119)
FMS	0.028(0.890)	0.072(0.726)	0.090(0.662)	0.519(0.013)	0.376(0.084)	0.519(0.013)

AD: Alcohol dependence; TC: total corrects; TE: total errors; PE: perseverative errors; NPE: non-perseverative errors; CC: categories completed; FMS: failure to maintain set.

**Fig 4:**
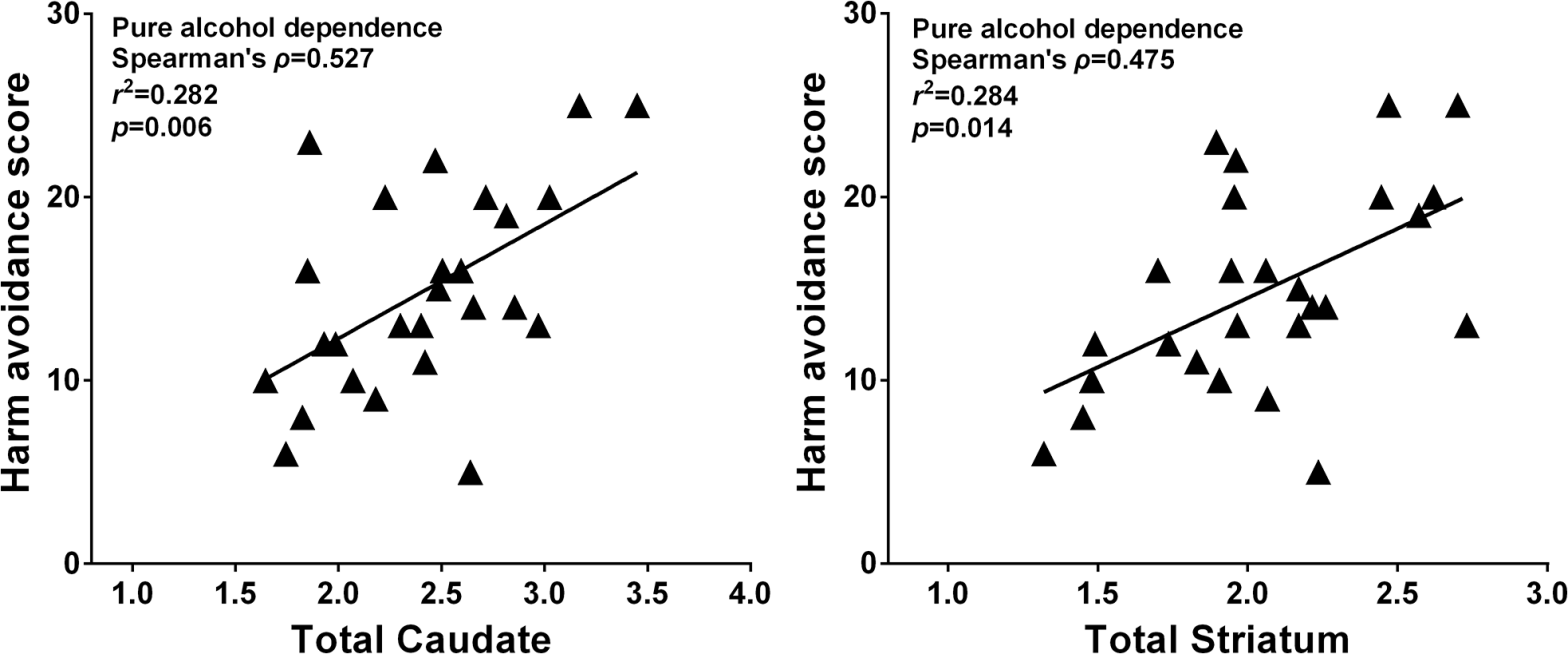
Graph showing correlation of striatal specific uptake ratio of [^99m^Tc]TRODAT-1with harm avoidance. Significant association between harm avoidance and SUR over total caudate(ρ = 0.527, *p* = 0.06) and total striatum (ρ = 0.475, *p* = 0.014) existed in pure alcohol-dependent individuals. The coefficient of determination (*r* ^*2*^) is 28.4% for the alcohol dependent group (*n* = 26) between harm avoidance and total striatum SUR.

Patients with AD demonstrated marginal differences from controls on novelty seeking (z = -1.723, p = 0.085) and harm avoidance (z = -1.973, p = 0.049) (Table 1). A significant positive correlation between harm avoidance and DAT availability was found in the patient group (rho = 0.475, p = 0.014 for striatum; rho = 0.474, p = 0.014 for the putamen; and rho = 0.527, p = 0.006 for the caudate, Conservative p value would be 0.05/3 = 0.017 after Bonferroni correction) (Fig 4), but not in the healthy control group (p > 0.05). The novelty seeking scale did not significantly correlate with DAT availability for either group in the brain regions studied.

## Discussion

### DAT availability in patients with alcohol dependence

DAT is widely expressed in the axons of dopamine neurons and its availability reflects maintenance of presynaptic function [27]. As hypothesized, the present study found a significant decrease of brain DAT availability in male patients with pure AD when compared to healthy controls. Our results are consistent with previous neuroimaging studies in patients with AD [7–9], but differ from others [10, 28].

There are three possible explanations for these conflicting results. First, alcohol dependence (or abuse) is usually co-morbid with anxiety disorder and/or depressive disorder [1], and evidence suggests the presence of higher DAT availability in depressive subjects than in healthy controls [29–31]. Therefore, the presence of patients with AD having comorbid anxiety/depressive disorders in a study group may be an important factor influencing findings of DAT availability, but previous brain imaging studies could not exclude these confounding factors [7–9, 28]. In order to exclude the influence of depressive factors on DAT availability, the present study enrolled subjects with pure AD only. Thus, false negative results due to inclusion of subjects with depressive disorders were unlikely.

Second, endogenous dopamine may compete with radioligands for the binding sites on DAT. Radioligands such as [^123^I]FP-CIT have been reported to compete with endogenous dopamine in an animal study [32]. If competition between endogenous dopamine and ^99m^Tc-TRODAT-1 indeed exists, we may have overestimated DAT availability in patients with pure AD because the extracellular dopamine level may decrease during alcohol withdrawal. Further studies should investigate whether ^99m^Tc-TRODAT-1 can be replaced by endogenous dopamine in humans.

Third, the timing of SPECT imaging during withdrawal or abstinence may influence the results of DAT availability in patients with AD. Evidence suggests that alcohol-related neurotoxicity in striatal dopaminergic neurons is reversible. A human study [8] administering [^123^I] β–CIT SPECT to subjects with alcohol dependence found a reduction in DAT availability during withdrawal followed by a significant increase with continued abstinence. This reversible neurotoxicity was also found in a previous animal study in which alcohol-preferring monkeys showed substantial improvement in alcohol-associated neurotoxicity and striatal DAT function after a period of abstinence [33]. In order to exclude the influence of a time effect on DAT availability, our patients were recruited during continued drinking behavior and the SPECT studies were performed within a stable withdrawal state (within 72 hours of the last drink). Thus, the time factor in abstinence from alcohol abuse is also unlikely to have interfered with our results.

### Effects of age, gender, brain regions and smoking status on DAT availability

Our results showed a significant negative correlation between striatal DAT availability and age in both patients with AD (r = -0.599, p = 0.001) and healthy controls (r = -0.573, p = 0.005), which is consistent with the findings of other studies [21, 34]. Previous studies using different radioligands have also shown decreased DAT availability related to age [35, 36]. However, our study showed no average age difference between patients with AD and healthy controls, thus eliminating age as a confounding factor in this study. With respect to gender effects, previous studies reported higher DAT availability in women [21, 31]; by enrolling only men, we excluded the effect of gender on DAT availability. The caudate and putamen are distinctly anatomically separate in the human, and we found that the DAT availability from greatest to lowest, in the three brain regions (ROIs) was caudate, striatum, putamen in both controls and patients. However, these brain regions were congruent reduction in DAT availability among patient group. These results suggested that the caudate and putamen may co-contributed to the development of patient with pure AD, but this suggestion should be further investigated.

Smoking may have indirect effects on the dopaminergic system [37]. Yang et al [38] failed to find the association [39]. The present study found no correlation between smoking and striatal DAT availability (rho = -0.217, p = 0.138), but the effect of smoking on DAT availability remains controversial and in need of further investigation with different clinical aspects.

### Cognitive functioning and dopamine dysfunction in AD

The WCST is one of the most important tools for assessing cortico-striatal circuit functioning and frontal lobe activity [40]. For instance, DAT knockout mice show diminished spatial cognitive functioning [41]. Alterations of dopamine levels within the basal ganglia may ultimately lead to cognitive deficits [42]. Additionlly, striatal dopaminergic neurotransmission modulates maintenance processes of working memory [43, 44]. In our study, Spearman's correlation analyses revealed a significant negative relationship between striatal DAT availability and total and perseverative WCST errors among the healthy groups (Fig 3). These results are in line with previous studies in humans [11] and experimental animals [45], suggesting that impaired dopaminergic functioning is associated with cognitive deficits and striatal DAT availability may play an important role in cognitive task performance. Although cognitive dysfunction and reductions in DAT availability were found among AD patients, only a marginally negative relationship between striatal DAT and total and perseverative errors was observed. One possibility for this result is that an early withdrawal state may influence this relationship among our pure AD patients; however, other possibilities should be further investigated.

Interestingly, but not surprisingly, no association was observed between striatal DAT availability and failure to maintain sets for both groups. Failure to maintain sets is defined as the process of making five or more consecutive correct choices followed by an error, without completing the category. Failure to maintain sets is regarded as a type of non-perseverative error [46]. In the present study, perseverative errors were possibly more sensitive than non-perseverative errors for identifying lower striatal DAT availability.

### Personality traits and DAT availability among patients with AD

The dopaminergic system and specific personality traits have been implicated in the development of AD [7, 16, 47]; however, the relationship between DAT availability and personality in AD has received very little attention. The present study found that patients with AD displayed higher NS and HA traits than healthy controls, in agreement with a previous study [16]. Additionally, the present study found that among patients with AD, high HA was related to high brain DAT availability (Fig 4). However, we could not find a significant correlation between NS and DAT availability for either group, even though a previous study speculated that NS behavior is positively related to DAT availability in antisocial AD [19]. Certain methodological differences may contribute to this discrepancy. For instance, the presents study used ^99m^Tc-TRODAT as the radioligand and selected a pure AD group (subjects having AD without comorbid Axis I or II disorders) from a Han Chinese sample. Conversely, Laine et al. [19] used [^123^I] β -CIT SPECT and selected an antisocial-subtype AD group from a European sample. Although these results support the idea that dopamine may play a role in regulating neuronal activity based on different personality types during AD development, specific methodological differences (e.g., different radioligands, populations, and AD subgroups) likely influenced divergent results. Therefore, the relationship between specific personality types and DAT availability should be further investigated using homogenous AD samples and subgroups as well as larger sample sizes.

### Limitations

The present study had some notable limitations. First, a cross-sectional design was used, AD is a complex disorder, and enrolled patients with pure AD could develop other mental illnesses later in life. This might be why our findings are perhaps discordant with other similar study. Second, our subjects with AD had taken lorazepam to prevent withdrawal symptoms before SPECT imaging, and there is evidence that benzodiazepines may interfere with levels of extracellular dopamine [48]. Third, the small sample size limited the power of our statistical analyses. To prevent possible Type I errors, we recruited only male patients with pure AD from a Han Chinese sample, thus limiting the effects of ethnicity and multifactorial diversity. Fourth, the present study focused on Cloninger’s original biosocial theory of NS and HA, but other personality traits such as persistence, self-directedness, cooperativeness, and self-transcendence were not assessed. Therefore, the study likely did not address all relevant personality traits (self-directedness, etc.) that could be associated with DAT availability in patients with AD.

## Conclusion

The present findings suggest that drinking behavior may influence the stabilization of brain dopamine levels and reduced striatal DAT availability. Low DAT availability may imply low dopamine levels in the brain, which would further influence neurocognitive deficits among patients with pure AD. Therefore, a reduction in DAT availability may play a pathophysiological role in the development of AD, which is also associated with neurocognitive deficits. Moreover, patients with pure AD who are high in trait–HA may show a positive relationship between DAT availability and the development of pure AD; however, different clinical subgroups and large-scale studies are needed to confirm this possibility.

## Supporting Information

S1 Table Specific uptake ratio in brain regions of pure alcohol dependent patients (ALC) and healthy controls. (DOCX)
