# Supplementary material for: Reduced Dopamine Transporter Availability and Neurocognitive Deficits in Male Patients with Alcohol Dependence
Source: PLoS One. 2015 Jun 29;10(6):e0131017. doi: 10.1371/journal.pone.0131017 (PMC4487997; doi:10.1371/journal.pone.0131017)
Supplement: S1 Table — (DOCX) [file pone.0131017.s001.docx]

**S1 Table** Specific uptake ratio in brain regions of pure alcohol dependent patients (ALC) and healthy controls.

| Brain area | Pure ALC | Healthy controls |  |  |
| --- | --- | --- | --- | --- |
|  | n = 26 | n = 22 | z | *p* -value^a^ |
| Lt Striatum | 2.02 ± 0.41 ^b^ | 2.56 ± 0.30 ^b^ | −4.201 | 2.654 x 10^-5^ |
| Rt Striatum | 2.08 ± 0.43 | 2.54 ± 0.30 | −3.622 | 2.929 x 10^-4^ |
| Lt Putamen | 1.66 ± 0.47 ^b^ | 2.21 ± 0.39 ^b^ | −3.882 | 1.038 x 10^-4^ |
| Rt Putamen | 1.74 ± 0.44 | 2.28 ± 0.37 | −3.805 | 1.182 x 10^-4^ |
| Lt Caudate | 2.41 ± 0.52 ^b^ | 2.88 ± 0.37 ^b^ | −3.135 | 1.716 x 10^-3^ |
| Rt Caudate | 2.42 ± 0.50 | 2.86 ± 0.40 | −2.939 | 3.295 x 10^-3^ |
| Total Striatum | 2.05 ± 0.39 | 2.55 ± 0.27 | −4.232 | 2.314 x 10^-5^ |
| Total Caudate | 2.41 ± 0.47 | 2.87 ± 0.32 | −3.249 | 1.160 x 10^-3^ |
| Total Putamen | 1.70 ± 0.42 | 2.25 ± 0.34 | −4.077 | 4.568 x 10^-5^ |

Lt = left ; Rt = right

All entries for brain specific uptake ratio in this table presented as mean ± SD.

^a^ *p* value of Mann-Whitney *U* test; Pure ALC Versus Controls.

^b^ There were no significant difference in DAT availability between left and right brain regions (including the caudate, putamen and striatum) in both subjects groups, separately.
